# Complex Effects of Hemp Fibers and Impact Modifiers in Multiphase Polypropylene Systems

**DOI:** 10.3390/polym15020409

**Published:** 2023-01-12

**Authors:** Denis Mihaela Panaitescu, Zina Vuluga, Adriana Nicoleta Frone, Augusta Raluca Gabor, Cristian-Andi Nicolae, Cătălina-Diana Uşurelu

**Affiliations:** Polymer Department, National Institute for Research and Development in Chemistry and Petrochemistry ICECHIM, 202 Splaiul Independentei, 060021 Bucharest, Romania

**Keywords:** polymer composites, hemp fibers, interface, multiphase system, impact strength

## Abstract

Natural fibers-reinforced polymer composites have progressed rapidly due to their undeniable advantages. Most of the commercial polypropylene (PP)-based materials are characterized by either high impact toughness or high stiffness, while the manufacture of PP composites with both good toughness and stiffness is challenging at present. In this work, poly[styrene-*b*-(ethylene-co-butylene)-*b*-styrene] (SEBS) and poly(styrene-*b*-butadiene-*b*-styrene) (SBS) copolymers were used in different amounts as modifiers in PP/hemp fibers (HF) composites, with the aim to use them for electrical vehicle parts. The interface in these multiphase systems was controlled by the addition of maleated polypropylene (MAPP). SEBS and SBS showed different effects on the elongation at break of the blends and the corresponding composites due to the HF that stiffened the multiphase systems. Similarly, a different action of MAPP was observed in the composites containing SEBS or SBS: higher Young’s and storage moduli were obtained for the composite containing SBS, while greater elongation at break and impact strength values were recorded for the SEBS-containing system. In addition, a remarkable dispersion in the MAPP-containing composite and two times smaller average particle size were revealed by the SEM analysis for the SEBS particles compared to the SBS ones. The higher affinity of SEBS for PP compared to that for SBS and the different morphological characteristics of the systems containing SEBS and SBS may explain the different effects of these impact modifiers on the mechanical properties of the composites. The composites developed in this work were designed as substitutes for the fully synthetic polymeric materials or metal components used in the manufacturing of automotive parts.

## 1. Introduction

Polymer composites have earned a good reputation over that of their metal counterparts due to their advantages of being lightweight, having good mechanical properties relative to their weight, a higher corrosion resistance, and easy processing in a wide variety of shapes and sizes [1,2,3]. Due to environmental concerns, natural fibers-reinforced polymer composites (NFPC) have seen faster progress in research and development than other polymeric composites have [3]. This visible progress has also been driven by the multitude of possible or demonstrated applications in automotive, construction, aircraft interiors, household appliances and, more recently, robotics [4,5,6,7].

Weight reduction is crucial in building pollution-free electric vehicles (EV). One possible route to reduce the EV weight is to increase the polymer/iron ratio from today’s 10–15% to over 40% [1], as just 10% reduction of the EV weight leads to a fuel economy of about 7% [8]. Due to their properties, NFPC can contribute to this target, while also enhancing the eco-friendliness of the design [9,10]. Expert Choice software and the Analytical Hierarchy Process method were used to select the optimal natural fiber-reinforced composite material to replace fully synthetic materials in the fabrication of automotive interior components [11]. The selection took into account several criteria such as the physical and mechanical properties, the environmental risk, along with the material and manufacturing costs. The polypropylene (PP)–hemp fibers (HF) composite was first in the ranking order, with it being selected as the optimal composite material for the manufacture of automotive components using greener technology [11]. PP/HF composites are also remarkable for their good specific properties, processability, and low cost [12,13]. Compared to other lignocellulosic fibers, HF are easier to grow, while they absorb large amounts of CO_2_ during cultivation, and they are characterized by outstanding mechanical properties relative to the density [14,15]. PP/flax or PP/HF composites are currently used in the manufacture of door panels, door bolsters, headliners, side and back walls, and other automotive parts by many manufacturers such as Mercedes-Benz, Chrysler, Ford, Honda, or Nissan [12,16]. Their contribution in the design of pollution-free EVs has stimulated new research on materials with better suitability for this application [17]. Although PP/natural fibers composites are recyclable at the end of the product life cycle, some aspects related to environmental durability, safety, and polymer and fiber degradation along with the issues related to the coexistence of petroleum- and bio-based polymer waste, such as the contamination risk and separation costs, still need to be addressed [18,19]. Previous studies have shown that the mechanical properties of PP/HF or PP/flax fiber composites remain unchanged after several reprocessing cycles; mechanical recycling is a promising route for the efficient and economical recovery of these wastes [20,21,22].

Most commercial PP products are characterized by either high impact toughness or high stiffness, while the manufacture of PP composites with both good toughness and stiffness is challenging at present [23]. Li et al. established a relationship between the composite’s modulus and the modulus of the modifier, content of modifier, modifier’s particle size, and interparticle distance based on Takayanagi’s two-phase model [23]. They showed that high impact PP composites with high stiffness are difficult to produce because the elastomer-toughened PP composites have a low critical interparticle distance of only 110 nm. They reported that the elastomeric modifier content has the most important effect on the modulus of the elastomer-toughened polymer composites [23].

Several attempts have been made to balance the stiffness and toughness in PP composites [24,25,26]. Yi et al. [24] obtained multiphase composites by adding wood particles (60 wt%) to a blend containing PP grafted with maleic anhydride (MAh) and an olefin block-copolymer (10 wt%). These composites showed an improved tensile strength and almost no reduction of the flexural modulus as compared to the PP/wood particles composite without elastomer and MAh grafting. Sudar et al. [25] used differently sized wood fibers (WF) as reinforcing agents for a PP homopolymer and a blend containing PP and maleated ethylene–propylene–diene copolymer (MAEPDM) as an elastomeric modifier. The composites also contained maleated polypropylene (MAPP) as a coupling agent. They observed an increase in the Young’s modulus (YM) in the three-component hybrid composites containing increasing amount of MAPP and WF, especially for the WF with the highest aspect ratio, and a decrease in the YM in the presence of MAEPDM [25]. The impact strength of these three-component hybrid composites decreased with increasing the WF content, regardless of the type of modifier or WF characteristics. Poly[styrene-*b*-(ethylene-co-butylene)-*b*-styrene] (SEBS) elastomer grafted with MAh (SEBS-MA) (20 wt%) was used as a compatibilizer in PP/wood flour composites with from 10 to 50 wt% wood flour [27]. The tensile strength and modulus of the composites increased with the wood flour concentration, while the elongation at break and impact strength strongly decreased. This decrease in deformability was explained by the increased number of stress concentration points that formed around the wood flour particles [27]. In another work, increasing the amount of SEBS-MA improved the impact strength of the PP/WF composites up to a content of 8 wt%, with the SEBS-MA acting as a compatibilizer and impact modifier [26].

In previous works, unmodified SEBS was used as an impact modifier in PP/HF composites containing MAPP as a compatibilizer [13]. It was observed that the addition of 15 wt% SEBS reduced the efficiency of the MAPP in the PP/HF composites [13], while the PP/surface-treated HF composites that were simultaneously modified with SEBS and MAPP showed improved mechanical properties [28]. In addition, the HF’s length and aspect ratio influenced the final properties of the PP composites containing 15 wt% SEBS [29]. However, a higher concentration of impact modifier in the presence or absence of MAPP could lead to more balanced PP/HF composites in terms of stiffness—toughness. Therefore, in this work, SEBS and poly(styrene-*b*-butadiene-*b*-styrene) (SBS) block-copolymer were used in different amounts as modifiers in the PP/HF composites intended for the manufacture of EV parts. MAPP was employed as a coupling agent to improve the interface and dispersion of the components in these PP—block-copolymer—HF multiphase systems. Both the PP blends and PP/HF composites with an increasing SEBS content and the blends and composites containing SBS as an impact modifier were obtained by melt extrusion and characterized in terms of the mechanical, thermal, and morphological properties. The composites developed in this work are designed as lightweight and cost-effective alternatives for the fully synthetic polymeric materials used in the production of automotive parts.

## 2. Materials and Methods

### 2.1. Materials

A polypropylene (PP) copolymer BJ380MO with a density of 0.906 g/cm^3^, a melt flow rate (MFR) of 80.0 g/10 min (230 °C/2.16 kg), and a melt processing temperature of around 200 °C was procured from Borealis AG (Vienna, Austria) and used as the polymer matrix. A linear poly[styrene-b-(ethylene-co-butylene)-b-styrene] (SEBS) copolymer with a styrene content of 29%, a number average molecular weight (M_n_) of 79,100 g/mol, a density of 0.91 g/cm^3^, a MFR of 5.0 g/10 min at 230 °C/5.0 kg, and a MFR<1.0 g/10 min at 200 °C/5.0 kg, which is commercialized as Kraton G1652, and a linear styrene-butadiene-styrene (SBS) block-copolymer with a styrene content of 30%, a vinyl content of 35%, a M_n_ of 138,000–162,000 g/mol, a density of 0.94 g/cm^3^, and a MFR<1.0 g/10 min (200 °C/5.0 kg), which is commercialized as Kraton D1192 E, were obtained from Kraton Polymers (Houston, TX, USA) and used as impact modifiers. Maleic anhydride-grafted polypropylene (MA) with a density of 0.91 g/cm^3^ and a melting temperature of 157 °C was purchased from Crompton (Middlebury, CT, USA) under the trade name of Polybond 3200 and employed as compatibilizer. Hemp fibers (HF) with a purity of 97 wt%, lengths in the range of 15–20 cm, and a density of 1.48 g/cm^3^ were supplied by HempFlax Group B.V. (Oude Pekela, Netherlands) and utilized as reinforcing agents.

### 2.2. Preparation of Composites and Specimens

Prior to using them, the HF were cut to a length of about 5 mm in a laboratory mill with adjustable die, dried in an oven at 90 °C for 4 h to remove moisture, and then kept in a desiccator until they were used as reinforcing agents. Next, the PP-based composites containing 30 wt% HF were prepared via melt blending in a twin-screw extruder. For this, neat PP, PP/SEBS, PP/SBS, PP/MA/SEBS, and PP/MA/SBS formulations were mixed at ambient temperature for 30 min in a Turbula T2F rotating mixer (Basel, Switzerland), and the resulting mixtures were transferred into a twin-screw extruder Brabender DSE 20 (Brabender, Duisburg, Germany). To avert HF’s degradation due to the harsh conditions at the beginning of the extrusion process and ensure a good dosing as well as a homogeneous dispersion of the HF filler in the molten polymer matrix, the desired amounts of HF were fed separately in the extruder through a secondary feeder. The screw speed was set to 150 rpm, and the temperature profile along the barrel of the extruder starting from the feeding zone to the die was 155–160–165–165–170–170 °C. The filaments exiting the extruder were circulated through a cooling water bath and granulated using a Brabender pelletizer (Duisburg, Germany) at a take-off speed of 9–10 m/min. Pure PP, PP/SEBS, PP/SBS, PP/MA/SEBS, and PP/MA/SBS blends that were free of HF were processed in the same conditions and used as references. Next, the granulated blends and composites were dried in an oven at 80 °C for 4 h and injection molded into dumbbell-shaped tensile specimens with the dimensions set by ISO 527-2 (specimens type 1B) and bar specimens (80 mm × 10 mm × 4 mm) for the impact test using an Engel Victory VC 60/28TECH injection molding machine (Engel, Schwertberg, Austria) at 185 °C. The code names and compositions of the prepared blends and composites are summarized in Table 1.

### 2.3. Characterization

The morphological characteristics of the composite films such as the hemp fibers’ dispersion and the hemp fibers–polymer matrix interface were investigated by scanning electron microscopy (SEM). The analysis was performed using a Hitachi TM4000 plus microscope (Hitachi, Tokyo, Japan) at an accelerating voltage of 15 kV on the impact strength specimens that were fractured in liquid nitrogen and sputter coated with a 5 nm layer of gold.

The mechanical properties of the composites were measured using an INSTRON 3382 Universal Testing Machine equipped with a Bluehill 2 Software (Instron, Norwood, MA, USA) in accordance with the ISO 527 standard. The tensile strength determination was performed on five dumbbell-shaped test specimens of each sample and at a crosshead speed of 50 mm/min. The Young’s modulus determination was performed analogously, but at a crosshead speed of 2 mm/min. The notched Izod impact strength was measured according to ISO 180 using a Zwick HIT5.5 Pendulum Impact Testers (Zwick Roell AG, Ulm, Germany). Seven specimens were used for each sample.

The dynamic mechanical analysis (DMA) of the prepared composites was performed using a DMA Q800 equipment (TA instruments, New Castle, DE, USA) on duplicate rectangular specimens with the length × width × thickness of 60 mm × 10 mm × 4 mm over a temperature range from −85 to 150 °C and at a heating rate of 3 °C/min in the multi-frequency strain test mode by employing the dual cantilever mode of operation and a load frequency of 1 Hz. For each sample, the variation of the storage modulus (*E*′) and the mechanical loss coefficient (tan δ) as function of the temperature were recorded.

Differential scanning calorimetry (DSC) was carried out using Q2000 V24.9 equipment (TA instruments, New Castle, DE, USA). For this, duplicates of each sample, which were 13–14 mg in weight, were loaded into alumina pans, cooled at −90 °C, and equilibrated for 5 min, then heated to 210 °C and equilibrated for 5 min (first heating cycle), cooled again at −90 °C and equilibrated for 5 min (cooling cycle), and finally heated to 210 °C and equilibrated for 5 min (second heating cycle) at a heating/cooling rate of 10 °C/min under a helium flow rate of 25 mL/min. The melting temperature (T_m_) of the prepared composites was considered to be the peak temperature of the melting endotherm; the crystallization temperature (T_c_) was considered to be the peak temperature of the exothermic event from the cooling cycle. A relative degree of crystallinity (X_c_) of the analyzed samples was calculated from the DSC curves recorded during the second heating cycle by using the following equation [29,30]:

(1) $$X_{C}\left[\%\right]=\frac{\Delta H}{\Delta H_{0}\times {\;w}_{\mathrm{PP}}}\cdot 100$$

ΔH is the heat of fusion of the studied composite (J/g), ΔH_0_ is the heat of fusion of hypothetical 100% crystalline PP (207 J/g [31]), and w_PP_ is the hypothetical mass fraction of PP in the blend or composite. The w_PP_ was calculated assuming that commercial PP is 100% PP because the actual additive content of this commercial PP sort is not known.

The thermogravimetric analysis of the prepared blends and composites was conducted using a TA-Q5000 IR device (TA instruments, New Castle, DE, USA) from room temperature to 700 °C at a constant heating rate of 10°C/min and a nitrogen gas flow rate of 40 mL/min. All of the measurements were performed in duplicate on samples of 12–14 mg.

The density of the polymer blends and composites (*ρ_exp_*, g/cm^3^) was estimated using a Kern ALT220 analytical balance (KERN & SOHN GmbH, Balingen-Frommern, Germany), with an accuracy of 0.0001 g, equipped with a density determination kit. Fragments from the injection molded samples were used for the measurements. The density was determined by Equation (2) [32]:

(2) $$\rho_{exp}=\frac{m_{air}\times d_{ethanol}}{m_{air}\;-\;m_{ethanol}}$$

In Equation (2), *m_air_*, *d_ethanol_*, and *m_ethanol_* represent the mass of the sample in air, the density of ethanol (0.78847 g/cm^3^), and the mass of the sample in ethanol. The density of injection molded blends and composites was determined as the average of three measurements.

The void fraction (*VF*, %) was calculated according to Equation (3) [33]:

(3) $$VF=\frac{\rho_{calc}-\rho_{exp}}{\rho_{calc}}\times 100$$

The theoretical value of density (*ρ_calc_*) was calculated using the rule of mixtures [24]:

(4) $$\rho_{calc}=\rho_{HF}\times V_{HF}+\rho_{m}\times \left(1-V_{HF}\right)$$

In Equation (4) *ρ_HF_* is the density of the HF (1.6 g/cm^3^ [34]), *V_HF_* is the volume fraction of the HF (0.21), which was calculated from the weight fraction of HF (0.3), and *ρ_m_* is the density of the polymer matrix (0.906 g/cm^3^).

## 3. Results and Discussion

### 3.1. Mechanical Properties

#### 3.1.1. Influence of Block-Copolymer Elastomers on PP Properties

**Figure 1:**
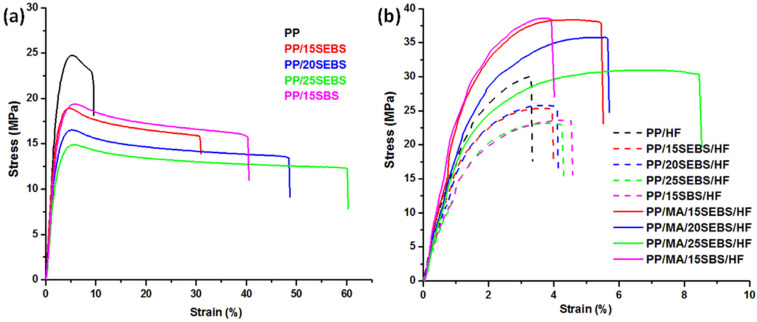
Stress–strain curves for the PP blends (**a**) and composites (**b**).

**Figure 2:**
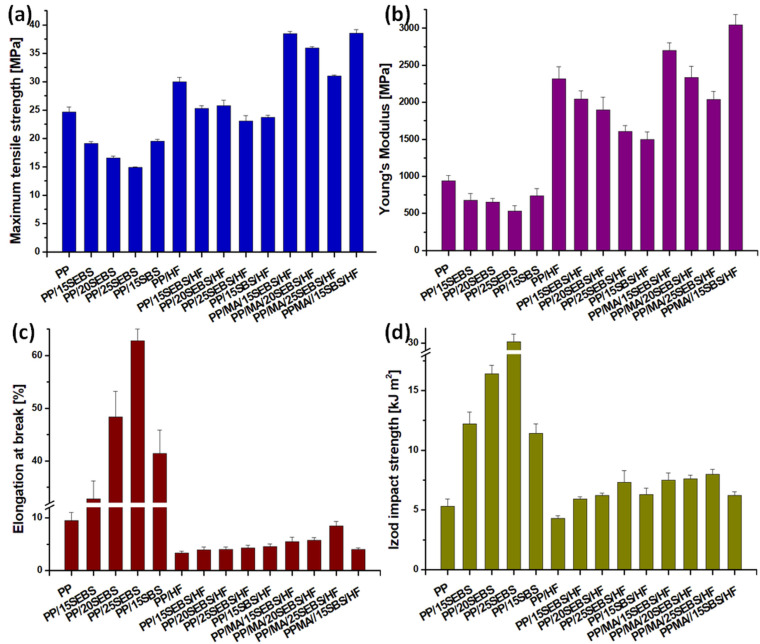
Tensile strength (**a**), Young’s modulus (**b**), elongation at break (**c**), and Izod impact strength (**d**) for the PP blends and composites.

The stress–strain curves of the PP blended with styrene block-copolymers are shown in Figure 1a. The addition of styrene block-copolymers strongly increased the deformability and toughness of the PP/SEBS or PP/SBS blends. Increasing the SEBS content in PP resulted in a seven-fold increase in the elongation at break and about three-fold increase in the energy at break for the PP blend containing 25 wt% SEBS. Detailed data regarding the tensile properties of the PP blends and composites are summarized in Figure 2. A six-fold increase in the impact strength was obtained for the PP blend with 25 wt% SEBS, and in general, a continuous increase in the impact strength with the amount of SEBS was observed for the PP/SEBS blends (Figure 2). This high increase in deformability was concomitant with reductions of the tensile strength and YM by 1.7 and 1.8 times, respectively. Although it is difficult to make a comparison with other published results due to the various sorts of PP, different preparation routes (in particular, melt mixing instead of extrusion processing and compression molding instead of injection molding), and different tensile test conditions, previous works have reported a similar or smaller increase in the elongation at break [35,36,37].

#### 3.1.2. Influence of HF and Compatibilizer in the Multiphase Systems

The stress–strain curves of the PP/HF composites containing different amounts of SEBS in the presence or absence of MAPP compatibilizer are shown in Figure 1b, and the detailed mechanical data are summarized in Figure 2. The addition of 30 wt% HF in the PP led to a three-fold reduction of the elongation at break, a 2.5-fold increase in the modulus, and a 30% increase in the tensile strength. A similar effect of the natural fibers in the PP matrix was previously signaled [13,38,39]. The impact strength of the PP/HF composite was only slightly affected by the presence of HF. The addition of increasing amounts of SEBS in the PP/HF composite led to a steady increase in the impact strength, which at a SEBS content of 25 wt% became almost twice that of the PP/HF sample, as well as to a steady decrease in the YM. The Young’s modulus of PP/25SEBS/HF, the composite with the highest amount of SEBS, was 1.7 times higher than that of the neat PP. The incorporation of 15 wt% SEBS into the PP/HF composite led to a 19% decrease in the tensile strength, while higher SEBS contents determined only a slight further decrease in the tensile strength of the composites.

Surprisingly, the elongation at break did not increase significantly by increasing the SEBS content in the composites. Unlike in the blends where the addition of 25 wt% SEBS led to a 560% increase in the elongation at break, the addition of SEBS in the composites led to a very small increase in the elongation at break. For the composite with the highest SEBS content (PP/25SEBS/HF), an elongation at break of only 4.3% was obtained, thus, it was half of that of the neat PP. A different effect of the thermoplastic elastomers on the elongation at break of blends and the corresponding composites was also reported by Denac et al. [36] for PP/silanized talc composites modified with SEBS. They observed that the elongation at break of the PP/SEBS blends increased strongly with an increasing SEBS content, while that of PP/talc/SEBS composites changed insignificantly. The reason behind this behavior is not quite clear at the moment. It can be assumed that the action of SEBS as a modifier is blocked in the PP/HF composites due to the rigid HF that restrict the mobility of the polymer chains in the PP and that of the ethylene-butylene (EB) domains of SEBS. Better adhesion between the HF and the PP/SEBS matrix should further restrict the deformation capacity of SEBS [40]. In addition, the changes in the adhesion at the PP–SEBS interface induced by the HF could also lead to larger SEBS domains, and thus, a smaller increase in the ductility of the composites [26]. Again, a larger affinity of HF for SEBS than that for PP may determine a preferential location of the HF near the SEBS domains of the multiphase systems, thereby reducing their ability to increase the ductility.

The addition of the MAPP compatibilizer in the PP/SEBS/HF composites led to a strong increase in the tensile strength and modulus. Thus, the tensile strength and YM have increased by 52 and 32% in the case of PP/MA/15SEBS/HF compared to those of the PP/15SEBS/HF composite, and by 34 and 27% for PP/MA/25SEBS/HF compared to those of the PP/25SEBS/HF sample, respectively. Similarly, the impact strength increased by 27 and 10% for the composites with 15 and 25 wt% SEBS, respectively, while the increase in the elongation at break was higher, of 40 and 100% for the same composites (Figure 2). Compared with the neat PP, the highest increase in the tensile strength and modulus was observed for the PP/MA/15SEBS/HF and PP/MA/15SBS/HF composites. Thus, the tensile strength increased by about 56% for both of the composites, while the increase in the YM was 188% for PP/MA/15SEBS/HF and 225% for PP/MA/15SBS/HF. Meanwhile, the impact strength increased by up to 50%, while the highest elongation at break value, which was obtained for PP/MA/25SEBS/HF, was close to that of the neat PP.

Small differences were observed between the effects of SBS and SEBS on the mechanical properties of the PP blends (Figure 2). Except for the impact strength, all of the other mechanical properties were slightly higher for PP/15SBS compared to those for PP/15SEBS. This is surprising since the choice of SEBS instead of SBS in these multiphase systems was generally based on an increased affinity between PP and SEBS due to the EB segments of SEBS. However, the advantage of SEBS over SBS in the PP/HF composites can be observed from the YM values: a 60% increase in the Young’s modulus was obtained for PP/15SBS/HF compared to that for the neat PP, while an almost double increase was achieved for PP/15SEBS/HF. On the contrary, in the MAPP-compatibilized systems, the addition of SBS led to a higher increase in the YM of PP (of 225% for PP/MA/15SBS/HF) compared to the SEBS-induced increase (of 188% for the PP/MA/15SEBS/HF multiphase system). This result supports previous observations [13] related to the action of MAPP as a compatibilizer at the PP–SEBS interface, which decreases its contribution at the PP–HF interface and leads to a lower increase in the YM. However, the PP/MA/15SEBS/HF composite showed a better elongation at break and impact strength compared to those of PP/MA/15SBS/HF.

**Figure 3:**
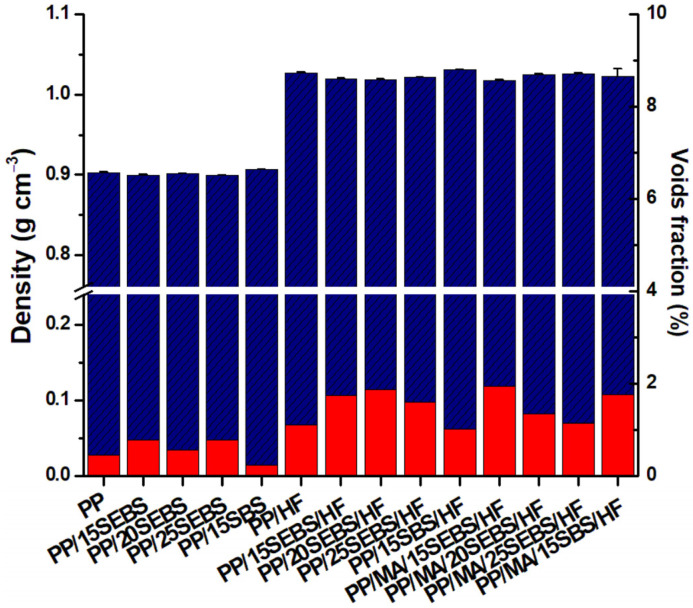
Density and voids fraction for the PP blends and composites.

The voids fraction was also determined, knowing that a higher voids fraction has a strong influence on the mechanical properties of the PP composites [41,42]. The presence of voids may be due to the poor impregnation of the fibers with a polymer or to the water carried by HF or additives, which are eliminated during melt processing, leaving empty spaces. Regardless of the origin, the voids decrease the mechanical properties of PP composites [41,42]. The voids fraction was almost twice as high in the composites compared to that in the blends, but it did not exceed 2% for any of the composites (Figure 3). Therefore, the presence of voids cannot explain the observed differences between the mechanical properties, especially considering that the impact strength and elongation at break of the composites are only slightly affected by voids fraction values lower than 2% [42].

### 3.2. Dynamic Mechanical Analysis

**Figure 4:**
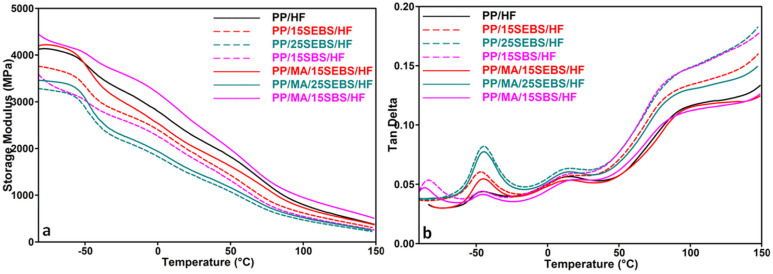
Storage modulus and tan δ vs. temperature profiles for PP/HF composites.

The variation in the mechanical properties with the temperature for the PP blends and composites was studied by DMA. Storage modulus (*E*′) and loss factor (tan δ) vs. temperature curves are presented in Figure 4 for the composites, while the DMA results for the blends and composites are given in Table 2.

The PP storage modulus was slightly influenced by the type of block-copolymer that was used as a modifier, and increasing the SEBS concentration in the blends led to an important decrease (of 20–40%) in the *E*′ of the PP (Table 2), which is in good agreement with the decrease in the YM of blends as determined by the tensile tests. The type of the block-copolymer had more influence on the storage modulus of the composites: the SBS impact modifier had a lower efficiency compared to that of SEBS in the PP/HF composites, but it had a higher efficiency than SEBS did in the composites containing MAPP. Thus, higher *E*′ values were obtained for PP/MA/15SBS/HF than for PP/MA/15SEBS/HF on the whole temperature range, thereby showing the lower efficiency of SEBS in the presence of MAPP. A similar result was obtained from the tensile tests, and it has been signaled in a previous study [13]. The different effects of SEBS and SBS in these multiphase polypropylene systems suggests a different action of MAPP and a possible location at the PP–SEBS interface compared to that for the PP–SBS one. SEBS has a higher affinity for PP than SBS does due to its EB blocks that can interdiffuse with the PP chains [43]. This may lead to the occurrence of smaller SEBS domains compared to the SBS ones in the multiphase systems, and therefore, to a larger interface. This increases the likelihood that MAPP is found at the PP–block copolymer interface in the PP/MA/15SEBS/HF system compared to the PP/MA/15SBS/HF one. As a result, the PP–HF interface in PP/MA/15SEBS/HF has less MAPP available to increase the compatibility, and therefore, to improve the mechanical behavior of the multiphase system. The increasing SEBS content in the composites decreased their storage modulus regardless of the presence of MAPP (Figure 4). However, the *E*′ values were higher for the PP/25SEBS/HF and PP/MA/25SEBS/HF composites compared to those for the PP reference, regardless of the temperature (Table 2).

The tan δ vs. temperature profile of the PP shows two glass transition temperatures at −47 °C (*T*′*_g_*) and 14 °C (*T_gPP_*) because the PP used in these experiments was a copolymer that probably contained ethylene, butylene, or octene segments. The addition of SEBS in the PP did not modify the position of these peaks due to the overlapping of the two relaxations, which correspond to *T_gSEBS_* and *T*′*_g_*, with the intensity of the first peak being greater in the blend. The intensity of tan δ peak has further increased in the blends and composites with higher amount of SEBS. A third relaxation peak was noticed at about 85 °C (*T_αPP_*); this was due to a lamellar slip and molecular chain rotation in the crystalline fractions of the PP [28]. The addition of HF slightly decreased the *T_gPP_*, and this trend was observed for most of the composites. This effect may be related to a slight increase in the free volume, which was determined by the addition of the HF. The presence of some residual moisture in the HF even after being dried at 90 °C for 4 h may also contribute to this effect.

Besides the α transition in the crystalline phase and the β transition in the amorphous phase of PP, the PP/SBS blend showed two glass transition temperatures, *T_gSBS_* at −87 °C, and *T*′*_g_*, the glass transition of the PP flexible segments, at −46 °C. These transitions did not change significantly in the composites probably because the adhesion between the components of these multiphase systems was not very high. However, the height of the peak corresponding to *T_gSEBS_* changed with the increase in the SEBS concentration due to the increased flexibility of the blends and composites (Figure 4). The effectiveness coefficient (*C*_90°C_) was determined according to [28] for estimating the influence of the impact modifier on the reinforcing effect of the HF, with a lower *C*_90°C_ value being correlated to a higher efficiency. Both SEBS and SBS decreased the reinforcing efficiency of the HF in the composites without MAPP, while in the composite series containing MAPP, the reinforcing efficiency of the HF was diminished only at the highest content of SEBS (Table 2).

### 3.3. Morphological Characterization

The different effects of SEBS and SBS on the mechanical behavior of the multiphase polypropylene systems were understood better after the morphological investigation. The morphologies of the selected blends and composites were studied by SEM. The addition of SEBS or SBS to the PP led to small changes in the aspect of the cryo-fractured surfaces (Figure S1). A better organization and a rougher surface, which indicates a tougher material, were observed in the SEM image of the PP/15SEBS blend compared to that of the PP/15SBS blend. Indeed, a slightly lower impact strength was revealed by the mechanical characterization for the blend containing SBS, however, a similar tensile strength and modulus were recorded for the two blends.

**Figure 5:**
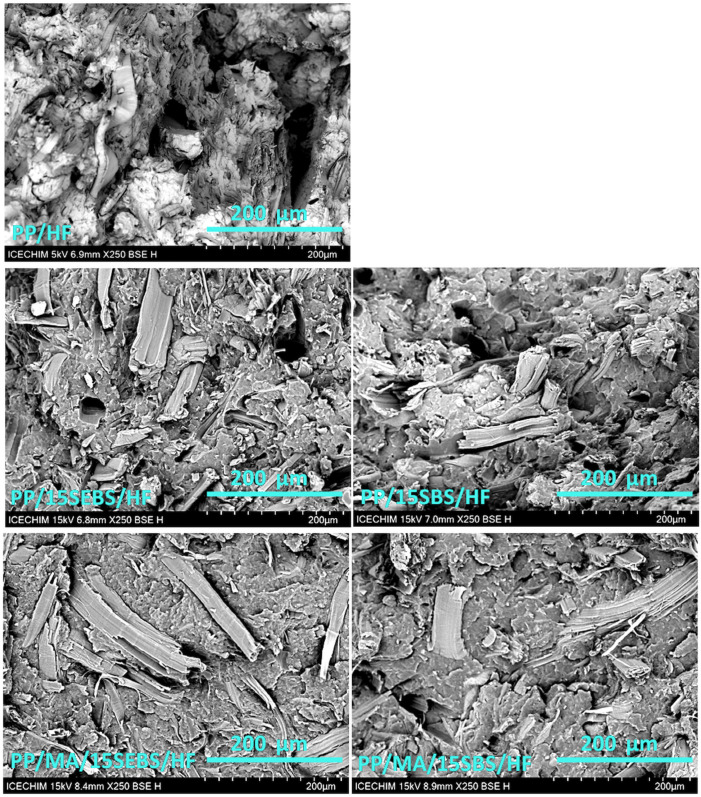
SEM images of fractured surfaces of composites (×250).

Low magnification SEM images of the selected composites are shown in Figure S2. These reveal a relatively good dispersion of the HF in all of the composites. The HF width varies over a wide range from tens of nanometers to 200 µm, with those that are below 100 µm being predominant. An average HF width of around 30 µm was determined from the SEM images of the composites, which is similar to other observations [29,44,45]. Higher magnification SEM images of the selected composites (Figure 5) provide information on how well these fibers are embedded into the PP or PP/SEBS (PP/SBS) matrices. The SEM image of PP/HF shows both the HF embedded in the PP matrix and numerous large holes which were left after fiber pulling during fracturing. The same situation was found in the PP/HF composites containing SEBS or SBS, with the difference being that fewer remaining holes and pulled fibers were observed. Another interesting aspect that was remarked in the SEM images of the block-copolymer-modified composites is the high number of sectioned fibers, indicating that the adhesion at the PP–HF interface was greater than the force required to break the fibers. Almost only broken fibers, which were mostly oriented transversally, were observed in the PP/HF composites containing MAPP and the block-copolymers, while holes were noticed only incidentally. Even when the fiber was pulled out, the remaining hole was not empty, but it contained fiber debris, indicating better adhesion at the PP–HF interface due to the MAPP coupling agent. This information on the fracture morphology explains the large increase in both the tensile strength and modulus that was observed in the composites containing MAPP.

**Figure 6:**
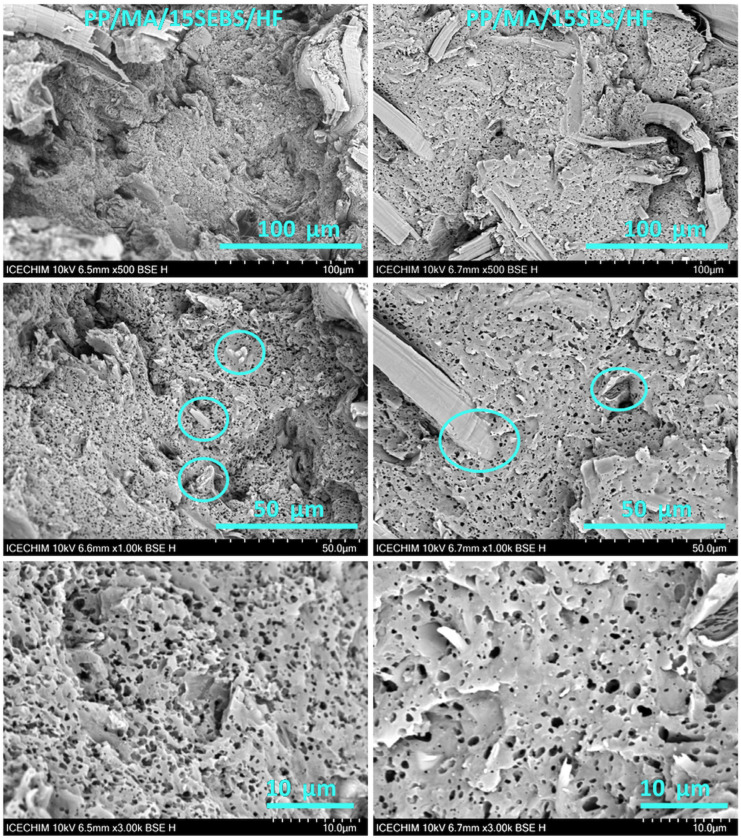
SEM images at different magnification of fractured surfaces of PP/MA/15SEBS/HF and PP/MA/15SBS/HF after etching.

Although the investigation of the composites by SEM provided valuable information, no obvious difference between the actions of SEBS and SBS was noticed. Therefore, the fractured surfaces from the PP/MA/15SEBS/HF and PP/MA/15SBS/HF composites were etched with tetrahydrofuran [35] at 50 °C for one hour to remove the block-copolymers, and they were dried at the same temperature for another 24 h before sputter coating them. The dispersion of SEBS and SBS in the sections of these composites was investigated by SEM. Figure 6 shows a remarkable dispersion in the section of the PP/MA/15SEBS/HF composite of the holes left after the SEBS extraction. Holes that were between 150 nm and 1800 nm in size and rare coupled holes resulting from the coalescence of SEBS particles were observed in the section of this sample. The average size of the holes, and thus of the SEBS particles in the PP/MA/15SEBS/HF composite, was 0.647 ± 0.410 µm, which makes them much smaller than the ones obtained using a single-screw extruder instead of the twin-screw extruder employed in this work, a different PP matrix, and no MAPP [35]. A very good dispersion of the SBS particles in the PP/MA/15SBS/HF composite was also remarked: holes with sizes ranging from 200 nm to 2300 nm were observed in the section of this composite. The average size of the SBS particles in the section of PP/MA/15SEBS/HF, which was determined by the average holes size in the SEM image, was 1.109 ± 0.574 µm, thus, it is almost double that of the SEBS particles. 

The smaller size of the SEBS particles further confirms the effect of the EB blocks on the compatibility between the PP and SEBS [35]. The smaller size of the SEBS particles in the PP/MA/15SEBS/HF composite compared to that of the SBS in the corresponding composite and the higher adhesion between SEBS and the PP matrix explain the better impact strength and elongation at break that were obtained for this composite (Figure 2c,d). However, the lower adhesion between the SBS particles and the PP matrix seems favorable to an increase in the YM, which is probably due to the fact that SBS induced a much lower flexibilizing effect on the PP compared to SEBS as a result of the weaker interface.

Another remarkable aspect is the very good adhesion between the HF and the polymer matrix, which covers the fibers in these composites (Figure 6: the circled areas are an example). No increase in the number of holes around the HF, but instead a homogeneous dispersion of them, was observed in the SEM images of PP/MA/15SEBS/HF, showing that the assumption of better adhesion of the HF with SEBS has no support as per the SEM images. More information on the effect of the HF and block-copolymers on the properties of these multiphase systems was obtained from the thermal analyses.

### 3.4. Melting and Crystallization Behaviors of Blends and Composites

**Figure 7:**
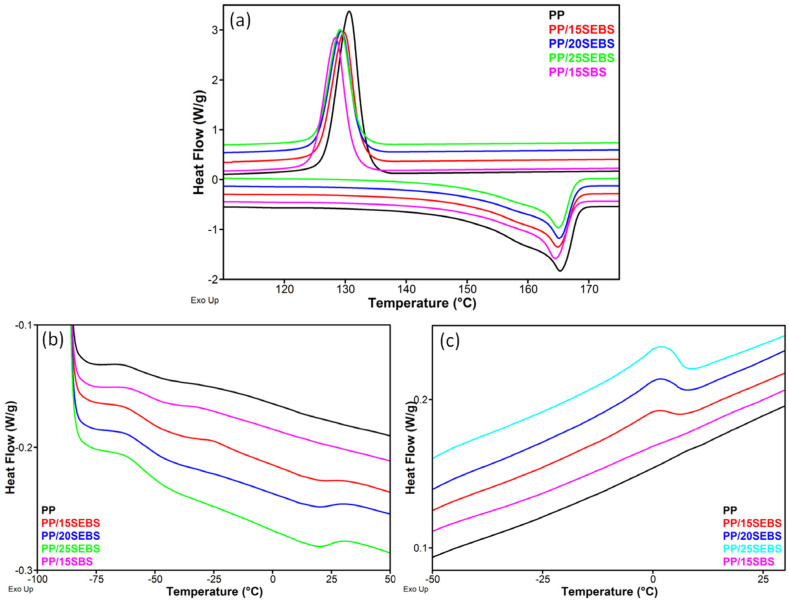
DSC melting and crystallization curves of the PP blends (**a**); melting curves showing the glass transition and melting of the EB blocks of SEBS (**b**); crystallization curves of the blends showing the crystallization of the EB blocks of SEBS (**c**).

The DSC curves from the second heating and cooling cycles of the PP blends are shown in Figure 7a, and the main characteristic data are summarized in Table 3. The slight decrease in the crystallization temperature (*T_c_*) and degree of crystallinity (*X_C_*) observed following the addition of SEBS or SBS in the PP shows that these modifiers disturbed the crystallization process of the PP to some extent. Marginal decreases in the degree of crystallinity and *T_c_* were also reported after the incorporation of 10 wt% SBS in the PP by melt mixing and compression molding [46]. A more significant decrease in *X_C_* and *T_c_* was reported for a PP/10 wt% SEBS blend that was obtained in similar conditions [47]. The addition of SEBS in the PP also leads to the occurrence of a melting process at around 18 °C (*T_mEB_*) and a glass transition at −55–−56 °C (*T_gEB_*), which are characteristic of the EB blocks (Figure 7b). Although the PP copolymer also showed a small transition at around −56 °C, which was determined by the content in elastomeric segments, the DSC cooling cycle showed only the crystallization of the EB blocks in the PP/SEBS blends at around 2 °C (Figure 7c).

**Figure 8:**
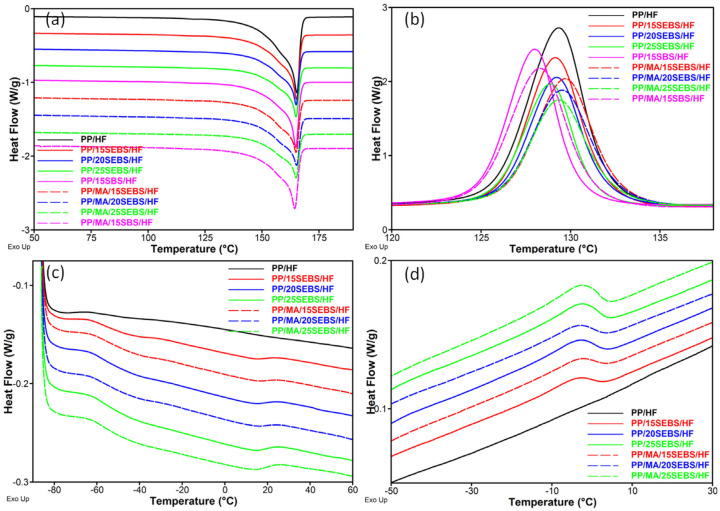
DSC curves of the composites: second melting cycle (**a**), cooling cycle (**b**), second melting cycle (SEBS temperature domain) (**c**), and cooling cycle (SEBS temperature domain) (**d**).

The melting and crystallization behaviors of the PP in the composites are disclosed in Figure 8. All of the composites showed melting and crystallization temperatures that are similar to those of PP/HF (Table 3).

Lower *T_c_* values were noticed for the composites containing SBS (Figure 8b), and slightly lower degrees of crystallinity compared to that of PP/HF were noticed for the PP/20SEBS/HF, PP/25SEBS/HF, and PP/15SBS/HF composites. However, all of the composites containing MAPP were characterized by a higher crystallinity than that for PP/HF (Table 3). The highest increase in the *X_C_* (13%) was calculated in the case of PP/MA/15SBS/HF, which also showed the highest increase in the YM. The increase in the *X_C_* value for the composites with larger amounts of SEBS after the addition of MAPP, was probably due to the coalescence of SEBS particles as their content increased [35], which decreased the size of the PP/SEBS interface and enhanced the MAPP effect of compatibilizing PP with HF, thereby increasing the crystallinity of PP. The higher *Xc* value obtained for the PP/MA/15SBS/HF was probably determined by the lower adhesion of SBS to the PP, leading to larger SBS domains and a higher compatibilizing effect of MA at the PP/HF interface, thereby increasing the *Xc*.

A decrease in the *T_mEB_* of 4–5 °C was observed in all of the composites compared to that of the blends (Figure 8c and Table 3). Similarly, a decrease in the crystallization temperature of the EB blocks (−2 °C) was observed in all of the composites compared to that of the blends (2 °C). It is worth remarking that the HF may add some moisture into the composites or lead to some degradative processes occurring in PP and SEBS(SBS). Furthermore, the addition of HF may disturb some SEBS–PP interactions through the formation of new PP–HF interactions that are favored by MAPP, thereby giving the SEBS chains greater mobility.

Therefore, a thermogravimetric analysis of the blends and composites was undertaken for assessing the effects of SEBS(SBS) and HF on the thermal stability of the composites.

### 3.5. Thermogravimetric Analysis of Blends and Composites

**Figure 9:**
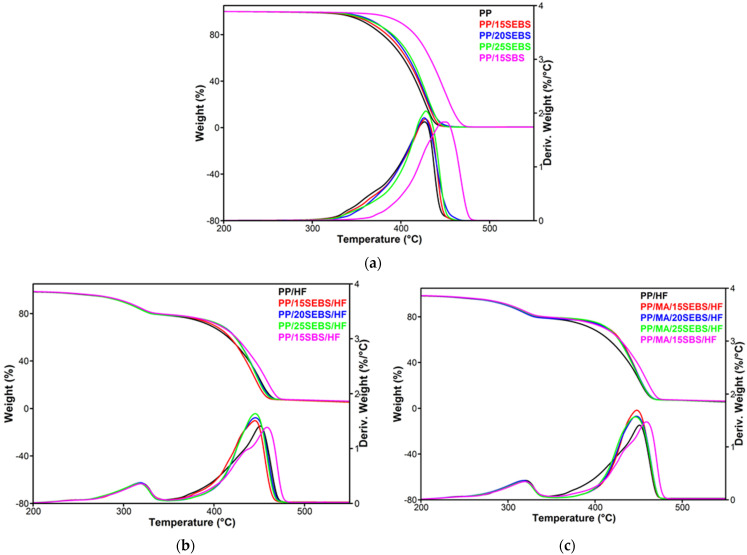
Thermogravimetric (TG) and derivative (DTG) curves for PP blends (**a**) and composites without MAPP compatibilizer (**b**) and with MAPP compatibilizer (**c**).

The thermogravimetric (TG) and derivative curves (DTG) of PP blends and composites are shown in Figure 9 and the TG–DTG results, such as the onset degradation temperature (*T_on_*), the temperature at 5% weight loss (*T*_5%_), the temperature at the maximum degradation rate (*T_max_*), and the weight loss at 200 °C (*WL*_200°C_) and the residue at 700 °C (*R*_700°C_), are disclosed in Table 4. The PP shows a single degradation step, with a *T_max_*_1_ at about 346 °C, although a small shoulder can be observed in the lower temperature range at around 350 °C. This may be due to the structure of the PP matrix, which is a copolymer. The addition of increasing amounts of SEBS led to an increase in the *T_on_* and *T*_5%_ of up to 10 °C, while the addition of 15 wt% SBS increased these characteristic temperatures by 30 °C and 40 °C, respectively. Meanwhile, the peak temperature (*T_max_*_1_) was shifted to a higher temperature by 24 °C for PP/15SBS. This large improvement in the thermal stability of the blends is due to the thermal behavior of SEBS and SBS, which lose less than 5% of their weight at up to 400 °C [48,49]. The addition of HF inserts a new peak (*T_max_*_2_) in the TG curves of the composites at around 318 °C. This is due to the thermal degradation of the HF which occurs at a lower temperature than that of the PP(*T_max_*_1_) at around 325 °C [28], which in this case is reduced to about 318 °C due to the fiber degradation during the composite manufacturing process.

The addition of increasing amounts of SEBS in the PP/HF composites did not have a significant effect on the thermal stability of the composites compared to that of PP/HF; this is probably due to the higher amount of friction induced during the melt process that was induced by the simultaneous presence of SEBS and HF, which may enhance the thermal degradation. However, the composite with 15 wt% SBS shows a slight increase in the characteristic temperatures, with the *T_max_*_1_ increasing by 8 °C compared to that of PP/HF (Table 4). Further, only a small increase in the *T_max_*_1_ was induced by the addition of the MAPP compatibilizer in the composites. 

It is worth noting that the influence of the SEBS(SBS) or the SEBS(SBS)+the MAPP compatibilizer on the thermal behavior of PP/HF composites is similar in some respects to that observed in the case of their mechanical properties, although the causes that determine these different behaviors are not the same. Thus, contrary to the case of blends, the addition of SEBS(SBS) or SEBS(SBS) + MAPP does not induce large changes in the thermal stability, elongation at break, or impact strength of the composites. However, the maximum degradation temperature was higher for all of the composites compared to that for the PP copolymer due to the effect of hemp fibers.

## 4. Conclusions

In this work, SEBS and SBS block copolymers were used as modifiers in the PP/HF composites intended for the manufacture of electrical vehicle parts. The addition of SEBS or SBS increased the elongation at break of the PP by up to 560% and decreased the strength and stiffness of the blends. The simultaneous addition of HF and SEBS strongly increased the tensile strength and the YM, and it slightly increased the elongation at break and impact strength, especially in the presence of MAPP compatibilizer. A different effect of the thermoplastic elastomers on the elongation at break of the blends and corresponding composites was observed as an effect of the HF, which stiffened the multiphase systems. Similarly, the enhancement of the thermal stability after the addition of SEBS(SBS) in the PP blends was not observed in the case of the composites, although the maximum degradation temperature was higher in all of the composites compared to that of the PP copolymer due to the effect of the HF. A different action of MAPP was also observed in the composites containing SEBS or SBS: a higher YM or storage modulus was obtained for PP/MA/15SBS/HF compared to that of PP/MA/15SEBS/HF, but a greater elongation at break and impact strength were achieved for the system containing SEBS. The higher affinity of SEBS when we were compared it to SBS for the PP, due to its EB blocks, led to smaller SEBS domains compared to those of SBS and to a larger interface that increases the possibility of finding MAPP at the interface between the block-copolymer and the PP in the PP/MA/15SEBS/HF system. This was demonstrated by the remarkable dispersion of the SEBS particles in PP/MA/15SEBS/HF and their smaller average size, which were two times smaller than they were in the case of SBS. The different morphological characteristics of the systems containing SEBS and SBS and the different adhesion of SEBS and SBS to the PP may explain the different effect of these impact modifiers on the mechanical properties of the multiphase systems. The composites developed in this work are designed as lightweight and cost-effective substitutes for the fully synthetic polymeric materials used in the manufacture of automotive parts.

## Figures and Tables

**Table 1:** Code names and compositions of the prepared blends and composites.

Samples	PP (wt%)	SEBS (wt%)	SBS (wt%)	MAPP (wt%)	HF (wt%)
PP	100	-	-	-	-
PP/15SEBS	85	15	-	-	-
PP/20SEBS	80	20	-	-	-
PP/25SEBS	75	25	-	-	-
PP/15SBS	85	-	15	-	-
PP/HF	70	-	-	-	30
PP/15SEBS/HF	55	15	-	-	30
PP/20SEBS/HF	50	20	-	-	30
PP/25SEBS/HF	45	25	-	-	30
PP/15SBS/HF	55	-	15	-	30
PP/MA/15SEBS/HF	50	15	-	5	30
PP/MA/20SEBS/HF	45	20	-	5	30
PP/MA/25SEBS/HF	40	25	-	5	30
PP/MA/15SBS/HF	50	-	15	5	30

**Table 2:** Storage modulus (*E*′) at selected temperatures; glass transitions of SEBS (*T_gSEBS_*), SBS (*T_gSEBS_*), PP (*T_gPP_*), and of the elastomeric segments in the PP copolymer (*T*′*_g_*); *T_αPP_* is characteristic to crystalline PP.

Samples	*E*′_−75_ GPa	*E*′_0_ GPa	*E*′_30_ GPa	*E*′_90_ GPa	*T_gSEBS_* °C	*T_gSBS_* °C	*T*′*_g_* °C	*T_gPP_* °C	*T_αPP_* °C	*C*_90°C_
PP	2.71	1.86	1.28	0.41	-	-	−46.8	13.9	84.8	
PP/15SEBS	2.64	1.68	1.14	0.34	−46.2	-	-	13.2	82.9	
PP/25SEBS	2.18	1.23	0.82	0.25	−44.6	-	-	14.3	82.7	
PP/15SBS	2.38	1.65	1.09	0.33	-	−87.0	−46.0	13.3	84.2	
PP/HF	4.13	2.80	2.17	0.93	-	-	−46.3	11.5	85.3	0.667
PP/15SEBS/HF	3.72	2.40	1.80	0.71	−47.5	-	-	11.9	82.0	0.742
PP/25SEBS/HF	3.26	1.83	1.35	0.54	−44.6	-	-	10.9	81.2	0.773
PP/15SBS/HF	3.40	2.27	1.69	0.63	-	−84.4	−46.2	10.9	80.0	0.789
PP/MA/15SEBS/HF	4.22	2.54	1.95	0.86	−45.9	-	-	11.4	87.5	0.660
PP/MA/25SEBS/HF	3.44	1.94	1.44	0.60	−44.0	-	-	11.9	82.2	0.733
PP/MA/15SBS/HF	4.31	3.19	2.44	1.08	-	−86.4	−45.3	12.5	83.4	0.632

**Table 3:** DSC results, glass transition of the EB blocks (*T_gEB_*), melting temperature of the EB blocks (*T_mEB_*), PP melting temperature and corresponding enthalpy from the second heating cycle (*T_mPP_*, ∆*H_mPP_*), PP crystallization temperature (*T_cPP_*), and the degree of crystallinity (*X_C_*).

Blends/Composites	*T_gEB_* °C	*T_mEB_* °C	*T_cPP_* °C	*T_mPP_* °C	∆*H_mPP_* J/g	*X_C_* %
PP	(−56)	-	130.6	165.3	94.9	45.8
PP/15SEBS	−54.8	18.0	129.8	164.9	75.2	42.7
PP/20SEBS	−56.0	18.2	129.4	165.1	71.3	43.1
PP/25SEBS	−55.6	18.5	129.2	165.0	66.3	42.7
PP/15SBS	(−52)	-	128.4	164.6	75.4	42.8
PP/HF	(−52)	-	129.3	165.3	67.3	46.5
PP/15SEBS/HF	−56.4	13.2	129.2	165.1	54.1	47.5
PP/20SEBS/HF	−56.1	12.6	129.3	165.0	45.9	44.3
PP/25SEBS/HF	−57.9	13.7	128.9	164.9	40.9	43.9
PP/15SBS/HF	(−52)	-	128.0	164.8	52.1	45.8
PP/MA/15SEBS/HF	−55.6	14.0	129.7	164.9	48.9	47.2
PP/MA/20SEBS/HF	−58.2	14.1	129.6	165.2	44.7	48.0
PP/MA/25SEBS/HF	−57.3	13.0	129.3	165.0	40.6	49.0
PP/MA/15SBS/HF	(−52)	-	128.3	164.3	53.7	51.9

**Table 4:** TG–DTG results.

Blends/Composites	*T_on_* °C	*T*_5%_ °C	*T_max_*_1_ °C	*T_max_*_2_ °C	*WL*_200°C_ %	*R*_700°C_ %
PP	383.7	345.6	426.7	-	0.14	0.6
PP/15SEBS	387.6	350.3	427.8	-	0.15	0.5
PP/20SEBS	389.2	357.6	426.3	-	0.11	0.4
PP/25SEBS	393.3	356.0	428.2	-	0.10	0.4
PP/15SBS	412.4	385.6	451.1	-	0.14	0.4
PP/HF	279.8	268.1	451.0	319.6	1.76	1.8
PP/15SEBS/HF	279.9	270.9	445.3	318.2	1.60	2.2
PP/20SEBS/HF	278.7	270.4	445.8	318.3	1.57	3.0
PP/25SEBS/HF	277.4	270.0	445.7	317.2	1.56	2.7
PP/15SBS/HF	280.0	273.1	458.6	319.2	1.51	2.9
PP/MA/15SEBS/HF	282.1	269.0	447.9	318.6	1.66	3.3
PP/MA/20SEBS/HF	280.2	268.9	447.5	317.7	1.56	3.1
PP/MA/25SEBS/HF	281.9	271.8	446.4	318.0	1.49	3.1
PP/MA/15SBS/HF	280.1	273.9	459.0	319.0	1.46	3.0

## Data Availability

The data presented in this study are available on request from the corresponding authors.

## Supplementary Materials

The following supporting information can be downloaded at: https://www.mdpi.com/article/10.3390/polym15020409/s1, Figure S1: SEM images of PP and its blends (PP/15SEBS and PP/15SBS); Figure S2: SEM images of fractured surfaces of composites.
