# Supplementary material for: Complex Effects of Hemp Fibers and Impact Modifiers in Multiphase Polypropylene Systems
Source: Polymers (Basel). 2023 Jan 12;15(2):409. doi: 10.3390/polym15020409 (PMC9865927; doi:10.3390/polym15020409)
Supplement: Supplementary file 1 [file polymers-15-00409-s001.zip › polymers-2142500-supplementary.pdf]

Complex Effects of Hemp Fibers and Impact Modifiers in Multiphase Polypropylene Systems

Denis Mihaela Panaitescu*, Zina Vuluga*, Adriana Nicoleta Frone, Augusta Raluca Gabor, Cristian-Andi Nicolae, Cătălina-Diana Uşurelu

Polymer Department, National Institute for Research and Development in Chemistry and Petrochemistry ICECHIM, 202 Splaiul Independentei, 060021, Bucharest, Romania; adriana.frone@icechim.ro (A.N.F.); raluca.gabor@icechim.ro (A.R.G.); cristian.nicolae@icechim.ro (C.A.N.); catalina.usurelu@icechim.ro (C.D.U.)

* Correspondence: panaitescu@icechim.ro (D.M.P.); zvuluga@icechim.ro (Z.V.);

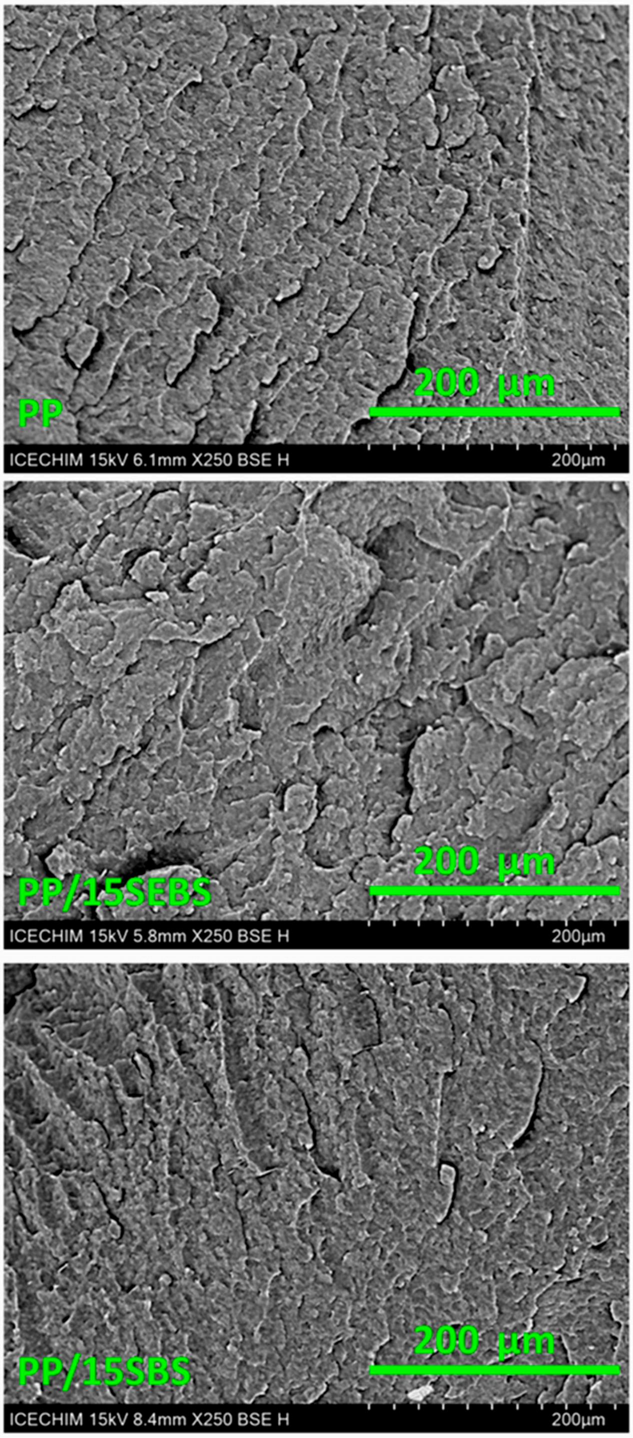

Figure S1. SEM images of PP and its blends (PP/15SEBS and PP/15SBS)

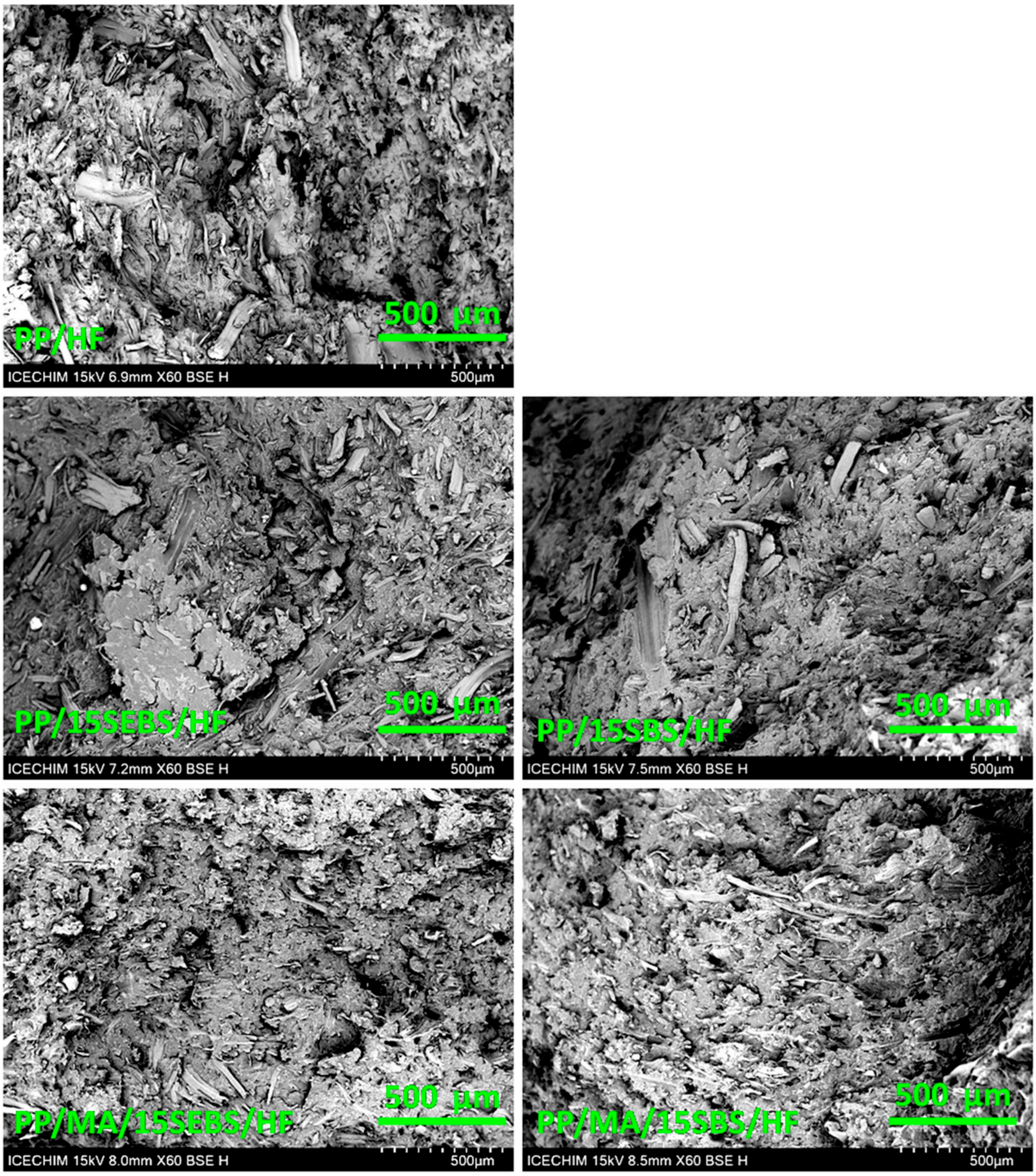

Figure S2. SEM images of fractured surfaces of composites
